# Anti-SRP and anti-HMGCR co-positivity in immune-mediated necrotizing myopathy with interstitial lung disease: A rare case report

**DOI:** 10.1097/MD.0000000000051232

**Published:** 2026-10-30

**Authors:** Muhammad Imran, Muhammad Hassaan Javaid, Zohad Fareh, Maryam Alam Wazir, Kamil Ahmad Kamil

**Affiliations:** a Department of Pulmonology, Shifa International Hospital, Islamabad, Pakistan; b Department of Medicine, Shifa College of Medicine, Islamabad, Pakistan; c Department of Medicine, Services Institute of Medical Sciences, Lahore, Pakistan; d Department of Medicine, Women Medical and Dental College, Abbottabad, Pakistan; e Department of Medicine, Nasrat Bashir Curative Hospital, Kandahar, Afghanistan.

**Keywords:** autoimmune myopathy, immune-mediated necrotizing myopathy (IMNM), interstitial lung disease (ILD), myositis, pulmonary involvement in myositis

## Abstract

**Rationale:**

Immune-mediated necrotizing myopathy (IMNM) is a rare inflammatory myopathy. Coexisting anti-signal recognition particle (anti-SRP) and anti-3-hydroxy-3-methylglutaryl-coenzyme A reductase (anti-HMGCR) antibodies in statin-naïve patients with interstitial lung disease are exceptionally uncommon.

**Patient concerns:**

A 55-year-old statin-naïve man presented with progressive proximal muscle weakness, dysphagia, myalgia, significant weight loss, and exertional dyspnea.

**Diagnoses:**

Serum creatine kinase was 8000 U/L. Anti-SRP, anti-HMGCR, and anti-Ro52 antibodies were positive. Chest computed tomography showed bilateral consolidations and ground-glass opacities suggestive of organizing pneumonia. Pulmonary function tests revealed reduced forced vital capacity (52%) and diffusing capacity for carbon monoxide (48%). Electromyography and muscle biopsy findings supported IMNM.

**Interventions:**

Treatment included high-dose corticosteroids, mycophenolate mofetil, nutritional support, and rehabilitation.

**Outcomes:**

After 3 months, muscle strength improved from 3/5 to 4+/5, dysphagia resolved, and weight increased by 5 kg. Creatine kinase decreased to 350 U/L, forced vital capacity improved to 74%, and diffusing capacity for carbon monoxide to 65%. Follow-up high-resolution computed tomography showed pulmonary improvement without fibrosis.

**Lessons:**

This case highlights the rare coexistence of anti-SRP and anti-HMGCR antibodies in statin-naïve IMNM with interstitial lung disease. Early immunosuppression, nutritional support, and rehabilitation may improve muscular and pulmonary outcomes. Comprehensive respiratory assessment and longitudinal monitoring are essential.

## 1. Introduction

Idiopathic inflammatory myopathies are a heterogeneous group of autoimmune disorders that include progressive muscular weakness and inflammation of the skeletal muscles. They include 5 distinct subgroups: dermatomyositis, polymyositis, nonspecific or overlap myositis, immune-mediated necrotizing myopathy (IMNM), and sporadic inclusion body myositis.^[1]^ The proportion of IMNM among all idiopathic inflammatory myopathies in Europe is 20% to 38%. A subgroup of these cases consists of anti-SRP antibody-associated myopathy and involves rapidly progressive proximal muscle weakness and highly elevated levels of creatine kinase (CK) in the absence of skin manifestations.^[2,3]^

While skeletal muscles remain the predominant target, a poor prognosis is frequently associated with extramuscular symptoms such as interstitial lung disease (ILD), dysphagia, and heart involvement.^[4–6]^ Interstitial lung disease is rare for anti-SRP myopathy but can involve organizing pneumonia or nonspecific interstitial pneumonia. In these cases, a multidisciplinary approach including rheumatology, neurology, and pulmonology becomes crucial.^[7,8]^

In this paper, we describe an extremely rare case of IMNM associated with anti-signal recognition particle (anti-SRP) and anti-3-hydroxy-3-methylglutaryl-coenzyme A reductase (anti-HMGCR) antibodies with weight loss and interstitial lung disease in a 55-year-old man. This case highlights the diagnostic challenges of this rare but clinically significant overlap presentation and the value of integrated multidisciplinary management.

This case report is written according to the CARE guidelines.^[9]^

## 2. Case presentation

A 55-year-old man with a history of cigarette smoking presented to the pulmonology outpatient department with progressive shortness of breath and exertional dyspnea. He had been diagnosed with ILD approximately 3 weeks earlier. Over the preceding 2.5 to 3 months, he had developed generalized myalgia, progressive proximal lower-limb weakness, dysphagia, and substantial unintentional weight loss of approximately 16 to 18 kg. He denied fever, hemoptysis, and night sweats.

His medical history was notable for gastroesophageal reflux disease (GERD), for which he had received intermittent treatment, as well as previous evaluation for weight loss and dyspepsia. He had no history of coronary artery disease or previous surgery and had never received statin therapy.

### 2.1. Investigations

Initial laboratory investigations demonstrated markedly elevated muscle enzymes, including CK and lactate dehydrogenase, with a serum CK level of 8000 U/L. Antinuclear antibody testing was strongly positive at a titer of 1:1280. Myositis-specific and myositis-associated antibody testing demonstrated positivity for anti-SRP, anti-HMGCR, and anti-Ro52 antibodies. Anti-mitochondrial antibodies were weakly positive.

Antinuclear antibody testing was performed by indirect immunofluorescence using human epithelial type 2 cells, whereas myositis-specific, myositis-associated, and anti-mitochondrial antibodies testing was performed using immunoassays. Serum protein electrophoresis showed no evidence of dysproteinemia. Screening for hepatitis viruses, human immunodeficiency virus, and tuberculosis was negative. Thyroid function tests were within normal limits.

Contrast-enhanced computed tomography (CT) of the chest, abdomen, and pelvis demonstrated bilateral peripheral and basal areas of consolidation with patchy ground-glass opacities, consistent with an organizing pneumonia pattern. There was no evidence of honeycombing, traction bronchiectasis, or established pulmonary fibrosis. A small mediastinal lymph node measuring 4 × 7 mm was noted without radiological features suggestive of malignancy. No pulmonary embolism was identified. CT of the abdomen showed no focal mass lesions involving the pancreas or liver.

Upper gastrointestinal endoscopy demonstrated a hiatal hernia with associated esophagitis. Pulmonary function testing showed a restrictive ventilatory defect, with reduced forced vital capacity (FVC) and total lung capacity, while the forced expiratory volume in 1 second/FVC ratio remained within the normal range. Diffusing capacity for carbon monoxide (DLCO) was moderately reduced, with no significant bronchodilator response. These findings, together with the clinical presentation and high-resolution computed tomography (HRCT) abnormalities, supported the diagnosis of ILD.

Neuromuscular investigations were performed because of the progressive proximal weakness, dysphagia, and markedly elevated CK level. Nerve conduction studies showed normal distal latencies, sensory and motor nerve action potential amplitudes, conduction velocities, and F-wave latencies, with no evidence of an axonal or demyelinating peripheral neuropathy. Needle electromyography (EMG) demonstrated short-duration, low-amplitude, polyphasic motor unit potentials with prominent myofiber irritability, supporting a primary myopathic process.

A muscle biopsy obtained from the right deltoid muscle showed scattered necrotic and regenerating muscle fibers at different stages of injury, with minimal inflammatory infiltration consisting predominantly of endomysial macrophages. Lymphocytic infiltration was absent. There was no evidence of perifascicular atrophy, vasculitis, granuloma formation, or inclusion bodies.

### 2.2. Diagnostic assessment

The diagnosis of IMNM was established based on the combination of subacute progressive proximal muscle weakness, dysphagia, markedly elevated serum CK levels, myopathic findings on EMG, characteristic muscle biopsy findings, and positivity for anti-SRP and anti-HMGCR antibodies. The concurrent diagnosis of ILD was supported by the characteristic HRCT findings and restrictive pulmonary physiology with reduced DLCO.

### 2.3. Management

The patient was managed by a multidisciplinary team comprising rheumatologists, neurologists, and pulmonologists. High-dose oral prednisone was initiated at 1 mg/kg/d. Given the severity of muscle involvement, dysphagia, ILD, and serological findings, mycophenolate mofetil was initiated at 1000 mg twice daily as steroid-sparing immunosuppressive therapy. Supportive management included nutritional rehabilitation and swallowing therapy.

### 2.4. Outcome and follow-up

The patient demonstrated marked clinical and biochemical improvement following initiation of prednisone and mycophenolate mofetil. After 3 months of follow-up, proximal muscle strength in both lower limbs improved from 3/5 to 4+/5 according to the Medical Research Council scale, and he was able to walk independently. Dysphagia resolved completely, and he gained 5 kg with improved nutritional intake.

Serum CK levels decreased substantially from 8000 to 350 U/L. Mycophenolate mofetil 1000 mg twice daily was well tolerated, with no reported gastrointestinal adverse effects, cytopenias, or infections. Prednisone was subsequently tapered according to the clinical response.

Respiratory symptoms and pulmonary function also improved. At 3 months, FVC increased from 52% to 74% and DLCO from 48% to 65%. Follow-up HRCT demonstrated substantial regression of the bilateral basal consolidations and ground-glass opacities, without evidence of pulmonary fibrosis or traction bronchiectasis. The patient remains under multidisciplinary follow-up with rheumatology, neurology, and pulmonology, with plans for continued steroid-sparing immunosuppressive maintenance therapy.

## 3. Discussion

This case represents an unusual presentation of immune-mediated necrotizing myopathy characterized by concurrent anti-SRP and anti-HMGCR antibody positivity in a statin-naïve patient with associated ILD. Co-positivity for these 2 antibodies is exceptionally uncommon and has been reported only rarely in the literature. The presence of ILD further expands the clinical spectrum of this uncommon serological phenotype.

IMNM is characterized histopathologically by necrosis and regeneration of muscle fibers with relatively limited inflammatory infiltration, distinguishing it from other idiopathic inflammatory myopathies such as dermatomyositis and polymyositis.^[10,11]^ Clinically, patients typically present with progressive proximal muscle weakness and markedly elevated serum CK levels. Dysphagia and extramuscular manifestations, including ILD, may also occur.

Characteristic features of IMNM include muscle weakness and significantly elevated CK. Coexistence of anti-SRP and anti-HMGCR antibodies without any previous exposure to statins is extremely rare and has only been reported in 1 individual in a case report,^[12]^ but we have not seen any other cases of coexistence of these antibodies with ILD. The presence of anti-HMGCR antibodies without any exposure to statins is rare and needs to be studied further for the exact mechanism.^[13–18]^

Individuals who test positive for anti-SRP usually experience increased severity of muscle symptoms, an elevated risk of atrophied muscles and difficulty swallowing, as well as markedly elevated levels of CK. Neoplasm association in individuals with the anti-SRP phenotype is very rare; however, 1 has to take into account the possibility of ILD, which is the case in this individual.^[19]^ In 17% to 20% of cases of IMNM patients suffering from ILD, there was an anti-SRP antibody.^[20–22]^ It has been suggested that smoking increases not only the susceptibility but also the severity of ILD in myositis due to oxidative stress caused by smoking and endothelial damage^[14]^; it is possible that smoking could increase disease severity in this patient, although a causal relationship cannot be established from a single case.

Hiatal hernia is an accepted risk factor for GERD, causing a reduction in lower esophageal sphincter pressure, more frequent transient lower esophageal sphincter relaxations, and impairment of esophageal clearance.^[23–25]^ Some previous publications about non-myositis ILD suggested the idea of silent microaspiration of the refluxed substances, especially in the presence of hiatal hernia, as possible reasons for the development of lung parenchymal damage^[26]^; however, without any proof of microaspiration on imaging and bronchoscopy, this suggestion is purely hypothetical and therefore the causal relation between GERD or microaspiration and this ILD cannot be confirmed.

Viral infection is considered one of the possible environmental triggers for IMNM since there were reported cases of disease development after viral illness, which means that viral antigens could cause immune system dysfunction in some patients.^[1,27]^ The absence of signs of viral infection in this patient makes this hypothesis less probable, but still possible due to retrospective analysis. It shows once again the complexity of IMNM pathogenesis, which includes immune injury due to autoantibodies, drugs, and some environmental triggers.

IMNM can be diagnosed through proper clinical examination as well as specific serological and histopathological tests. Apart from weakness that occurs acutely or subacutely, patients can have symptoms such as proximal weakness, dysphagia, weight loss, and heart and lung complications.^[28]^

Treatment for IMNM involves immunosuppressive medications because, just like other systemic idiopathic inflammatory myopathies except for inclusion body myositis, IMNM requires similar medical intervention. High-dose steroids should be used to treat IMNM right away due to their general effectiveness. According to an ENMC workshop, the initial treatment for IMNM should involve the use of both steroids and methotrexate.^[1,29,30]^

## 4. Conclusion

This case describes rare concurrent anti-SRP and anti-HMGCR antibody positivity in a statin-naïve patient with immune-mediated necrotizing myopathy and associated interstitial lung disease. The diagnosis was supported by the clinical presentation, markedly elevated CK level, myopathic EMG findings, characteristic muscle biopsy features, and dual myositis-specific antibody positivity. The patient showed substantial clinical, biochemical, and pulmonary improvement following immunosuppressive therapy. Although smoking and GERD with possible microaspiration were present, their contribution to the pulmonary findings cannot be determined from this case. Further reports and studies are needed to clarify the clinical significance of anti-SRP/anti-HMGCR co-positivity and its potential association with pulmonary involvement.

## Author contributions

**Conceptualization:** Muhammad Imran.

**Resources:** Maryam Alam Wazir.

**Supervision:** Kamil Ahmad Kamil.

**Writing – original draft:** Muhammad Hassaan Javaid.

**Writing – review & editing:** Zohad Fareh, Kamil Ahmad Kamil.
